# Application of multimodal analgesia combined with opioid-free anesthetics in a non-intubated video-assisted thoracoscopic surgery bullectomy: A case report

**DOI:** 10.3389/fsurg.2023.1116523

**Published:** 2023-02-13

**Authors:** Longbin Zheng, Xiaojing Zhang, Qing Ma, Weimin Qin, Wenbo Liang, Zhiqiang Ren, Guoxiang Fan, Ning Yin

**Affiliations:** Department of Anesthesiology, Sir Run Run Hospital, Nanjing Medical University, Nanjing, China

**Keywords:** non-intubated video-assisted thoracoscopic surgery (NIVATS), thoracic paravertebral nerve block (TPVB), enhanced recovery after surgery (ERAS), asthma, opioid-free, case report

## Abstract

**Background:**

Non-intubated video-assisted thoracoscopic surgery (NIVATS) has been increasingly applied worldwide owing to its benefits of enhanced recovery after surgery (ERAS). Anesthetic management for patients with asthma should focus on minimizing airway stimulation.

**Case description:**

A 23-year-old male patient with a history of asthma was diagnosed with left-sided spontaneous pneumothorax. The patient then underwent left-sided NIVATS bullectomy under general anesthesia with preserved spontaneous breathing. Left thoracic paravertebral nerve block (TPVB) with an injection of 0.375% ropivacaine (30 ml) was performed in the 6th paravertebral space under ultrasound guidance. Anesthesia induction commenced until the cold sensation in the surgical area had disappeared. General anesthesia was induced by midazolam, penehyclidine hydrochloride, esketamine, and propofol and then maintained using propofol and esketamine. Surgery commenced after the patient was positioned in the right lateral recumbency. The collapse of the left lung was satisfactory, and the operative field was ensured after artificial pneumothorax. The surgical procedure was uneventful, intraoperative arterial blood gases were within normal ranges, and vital signs were stable. The patient awakened rapidly without any adverse reactions at the end of the surgery and was then transferred to the ward. During the postoperative follow-up, the patient experienced mild pain 48 h after surgery. The patient was discharged from the hospital 2 days postoperatively and developed no nausea, vomiting, or any other complications.

**Conclusion:**

The present case suggests the feasibility of TPVB in combination with non-opioid anesthetics to provide high-quality anesthesia in patients undergoing NIVATS bullectomy.

## Introduction

Asthma is a common chronic inflammatory airway disease with increasing prevalence worldwide (1). Asthmatic patients who underwent surgery had a higher risk of perioperative morbidity and mortality due to bronchospasm and hypoxemia (2). Therefore, the anesthetic management for patients with asthma presents a challenge for anesthesiologists, especially when general anesthesia with tracheal intubation is required (3). Furthermore, optimizations of anesthetic agent selection and airway management are crucial to guarantee the life safety of asthmatic patients. Non-intubated video-assisted thoracoscopic surgery (NIVATS) has been widely used worldwide because of its advantages of enhanced recovery after surgery (ERAS) (4). To minimize the perioperative risk of anesthetic management, NIVATS under spontaneous respiration might be an optimal therapeutic option for asthmatic patient who underwent thoracic surgery.

Here, we report a case of a 23-year-old male patient with a history of asthma underwent left-sided NIVATS bullectomy by multimodal analgesia combined with appropriate sedation. We would like to highlight that ultrasound-guided thoracic paravertebral nerve block (TPVB) in combination with optimum non-opioid anesthetics is feasible for NIVATS bullectomy by providing high-quality anesthesia.

## Case report

### Case description

**Figure 1:**
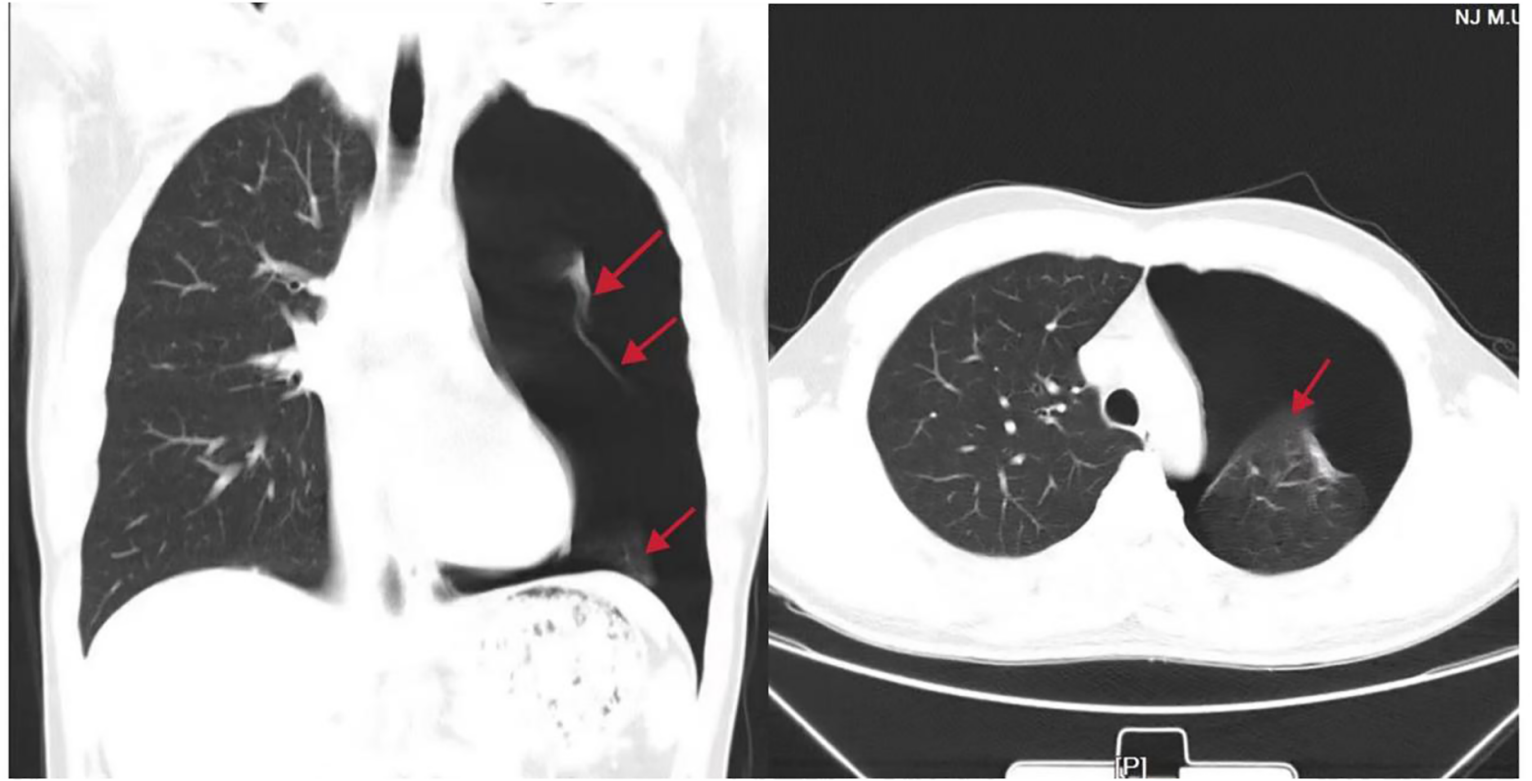
Preoperative chest radiograph and computed tomography scan showing an 80%–90% left-sided pneumothorax (red arrow).

A 23-year-old male patient (height: 175 cm, weight: 52 kg), American Society of Anesthesiologists II, presented to the hospital owing to acute left-sided chest pain and tightness and shortness of breath for 6 h. Chest CT performed on admission showed an 80%–90% left-sided spontaneous pneumothorax (Figure 1), and the patient was diagnosed with pneumothorax and administered nasal catheter oxygen and immediately treated with closed drainage of the thoracic cavity in the emergency room. The vital signs were monitored, NIBP 126/67 mmHg, HR 73 bpm, SpO_2_ 98%. The patient had a history of asthma for 12 years, well-controlled with regular use of albuterol spray, without any history of attack within 1 month. He self-reported a history of spontaneous pneumothorax 2 years ago, which recovered after conservative treatment. Otherwise he was healthy with no other disease history, and all preoperative laboratory and electrocardiogram findings were normal. The patient underwent left-sided NIVATS bullectomy under general anesthesia, with preserved spontaneous breathing after the completion of preoperative preparation.

On the day of the operation, the patient fasted for 8 h and did not receive any premedication. After entering the operating room, the venous channel of the right upper limb was opened, standard monitoring was established, and vital signs were within the normal range. Additionally, right radial artery catheterization was performed to monitor arterial blood pressure (ABP) and blood samples were collected. Vitals were recorded as follows: ABP 121/65 mmHg, HR 66 bpm, SpO_2_ 98%, and BIS 98. Arterial blood samples were obtained for blood gas analysis just before the operation, 15 min after artificial pneumothorax, at the end of the operation, and 15 min after awakening; the results are shown in Table 1.

**Table 1:** Perioperative arterial blood gas analysis data.

	Pre-operation	Fifteen min after artificial pneumothorax	End of operation	Fifteen min after awakening
pH	7.42	7.36	7.33	7.38
PaCO_2_ (mmHg)	38.3	44.2	48.7	39.5
PaO_2_ (mmHg)	133.3	134.8	151.8	154.5
BE (mEq/L)	2.4	1.6	0.9	1.3

Oxygen supplementation was administered using a mask at 2 L/min. The loading dose of dexmedetomidine (0.5 μg/kg) was infused intravenously within 10 min, Ramsay score was 3, and BIS value was 75–80 in the quiet state after dexmedetomidine administration. After the sterile technique, the left TPVB was performed under ultrasound guidance. A low-frequency convex array probe was placed in the 6th intercostal space and rotated to locate it in the 6th paravertebral space. A 22G 80-mm short beveled needle was advanced under ultrasound guidance using an in-plane technique to the 6th paravertebral space. After frequent aspiration to avoid intravascular injection, 30 ml of 0.375% ropivacaine was injected into the paravertebral space, and a high bright arc and dark drug area were visible on ultrasonography. The vital signs were stable, no complications were observed, and the cold sensation on the operative side was lost from 3rd to 8th thoracic nerve level approximately 20 min after the operation.

Anesthesia was induced until the cold sensation in the surgical area disappeared. General anesthesia was induced using midazolam 4 mg, penehyclidine hydrochloride 0.5 mg, esketamine 30 mg, and propofol 30 mg. After 5 min, the BIS value was 50–60, oxygen supplementation was administered by mask at 5 L/min, and the patient breathed smoothly and spontaneously. Subsequently, anesthesia was maintained using propofol 4–8 mg/kg/h and esketamine 0.5 mg/kg/h. BIS was maintained at 50–60, spontaneous respiratory rate was 15–18 breaths/min, and vital signs were stable.

Surgery commenced after the patient was positioned in the right lateral recumbency, and the thoracic surgeon placed a 2 cm incision in the 4th–5th intercostal space of the left anterior axillary line. After starting the artificial pneumothorax, the collapse of the left lung was satisfactory and operative field was ensured. Arterial blood gases were within normal ranges, and vital signs were stable. The surgical procedure was uneventful, without complications, such as respiratory depression, coughing, or body movement. The surgery lasted 40 min, with a bleeding volume of 5 ml, urine volume of 300 ml, crystallite infusion of 500 ml, and colloid infusion of 500 ml. After resecting the bullae, the lungs were slowly expanded using noninvasive positive pressure ventilation with a mask for 20 s. The infusion of esketamine and propofol was discontinued 15 min before the end of surgery. The patient regained consciousness immediately at the end of surgery and flumazenil (30 mg) was injected intravenously. He was instructed to nod his head 2 min after flumazenil administration, and the vitals were recorded as follows: ABP 112/63 mmHg, HR 59 bpm, SpO_2_ 96%, and BIS 96.

The patient was transferred to the PACU for monitoring, and the vital signs were stable. After 30 min of observation in the PACU, the patient was transferred to the ward. During the postoperative follow-up, the patient had a visual analog scale (VAS) score of 1 within 24 h after surgery, ≤2 at rest, and 3–4 during cough at 24–48 h after surgery. Postoperative chest CT showed that the left lung was inflated well, with no abnormalities. The patient was discharged from the hospital 2 days postoperatively and developed no nausea, vomiting, or any other complications.

## Discussion

In the present case, the patient was a 23-year-old man with a history of asthma who was otherwise healthy. Therefore, the anesthetic management in the present case focused on avoiding airway irritation, and choice of anesthetic drugs was more cautious and optimal. Herein, we report a case of multimodal analgesic management in combination with appropriate sedation that was successfully used to provide high-quality anesthesia in NIVATS bullectomy.

Video-assisted thoracoscopic surgery was routinely performed under endobronchial double-lumen intubation and single-lung ventilation along with general anesthesia. Although the surgical procedure is convenient with a clear operative visual field and is beneficial for surgery, the rate of complications such as airway injury is reportedly high (2). NIVATS strategies have become increasingly used worldwide because of less trauma, more comfort, and faster postoperative recovery in recent years (4). Patients with asthma commonly have a higher risk of bronchospasm during general anesthesia owing to airway inflammation and hyperreactivity (5). Hence, NIVATS was the best choice in the present case. Stable spontaneous breathing following adequate anesthesia is indispensable to prevent conversion to general anesthesia with tracheal intubation in NIVATS. Opioids, the most commonly used analgesics in general anesthesia, can frequently lead to respiratory depression. Therefore, multimodal analgesia by the synergistic use of non-opioid adjuncts and the regional nerve block technique was performed.

It is essential to provide adequate analgesia during NIVATS. Nerve blocks have been developed and proven feasible for opioid-free anesthesia applications. TPVB is achieved by injecting a local anesthetic into the thoracic paravertebral space, thus blocking the thoracic spinal nerve on the operative side (6). In this case, stress responses, such as coughing, body movement, and hemodynamic disturbance, did not occur during surgery. This is mainly attributed to the satisfactory analgesic effect provided by TPVB, thereby decreasing the intraoperative analgesic requirements.

To ensure intraoperative safety, anesthetic drugs that contribute to hemodynamic stability without respiratory depression such as dexmedetomidine, midazolam, and esketamine were applied in anesthesia induction. Anesthesia was maintained using esketamine and propofol. Spontaneous breathing was maintained during surgery. Although the intraoperative partial pressure of arterial carbon dioxide was higher than the normal range for some time, it returned to normal levels within a short period after surgery, and the patient woke up rapidly without any relevant complications.

Esketamine, a right-lateral dismantled fission of ketamine, is a novel NMDA antagonist that can provide similar unique dissociative anesthesia and displays an analgesic effect twice that of ketamine without respiratory depression (7). The application of ketamine is beneficial for ameliorating bronchoconstriction due to its sympathomimetic effect on bronchial smooth muscle tone (8). Because of its favorable effect on respiratory maintenance and synergistic anesthesia, esketamine was used in the present case. Intraoperative hemodynamic stability is closely associated with the sympathomimetic effect of esketamine. It was confirmed that the administration of ketamine frequently results in increased secretion production (9), which was not observed in the present study. This was primarily owing to the penehyclidine hydrochloride we used in anesthesia induction. Additionally, no adverse events, such as nausea, vomiting, or agitation, were experienced in this case. However, the VAS score at 24 h after the surgery was higher than before, which was mainly due to the analgesic effect gradually disappearing with local anesthetic metabolism after a single shot of TPVB.

## Conclusion

In conclusion, the present case suggests the feasibility of TPVB in combination with non-opioid anesthetics to provide high-quality anesthesia in a patient undergoing NIVATS bullectomy, which reminds us of more experience for better anesthesia management conforms to ERAS in NIVATS. However, opioids remain the primary and most commonly used analgesics in clinical practice, and whether this anesthetic protocol is applicable to more complex surgical procedures with a longer duration needs to be explored.

## Data Availability

The original contributions presented in the study are included in the article/Supplementary Material, further inquiries can be directed to the corresponding author.
